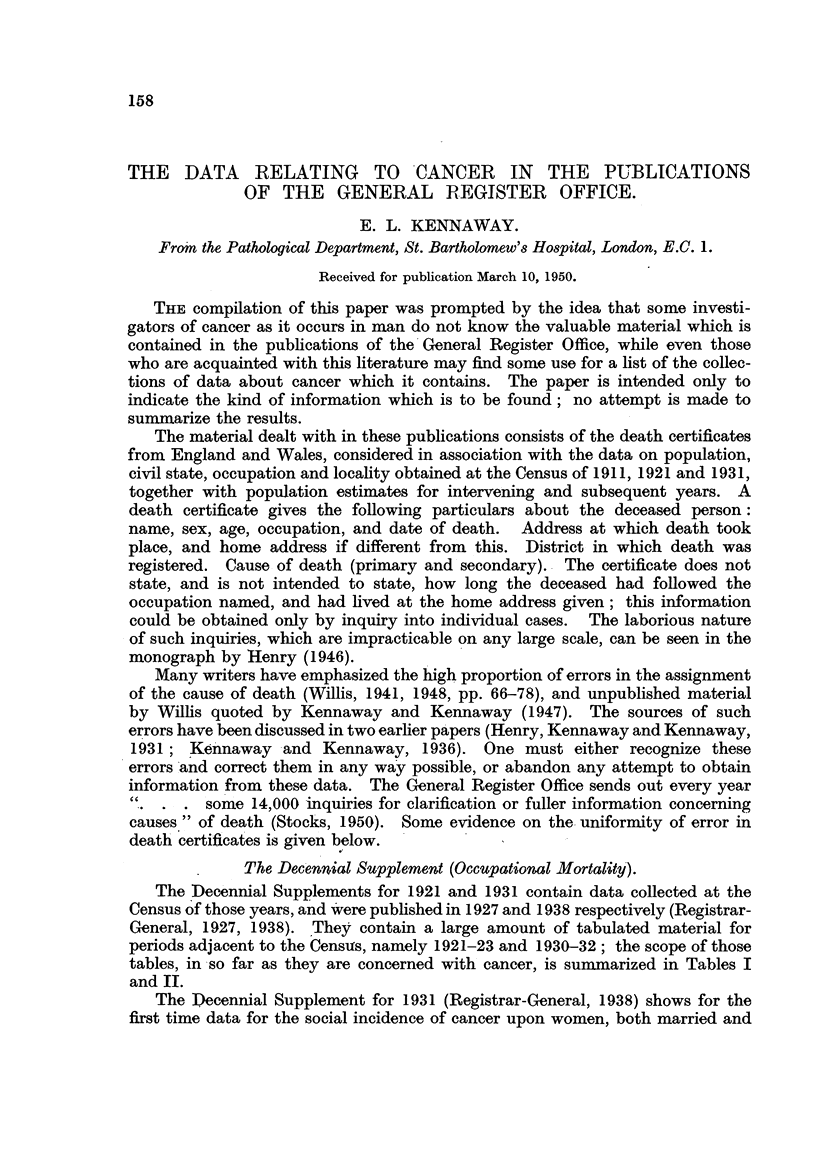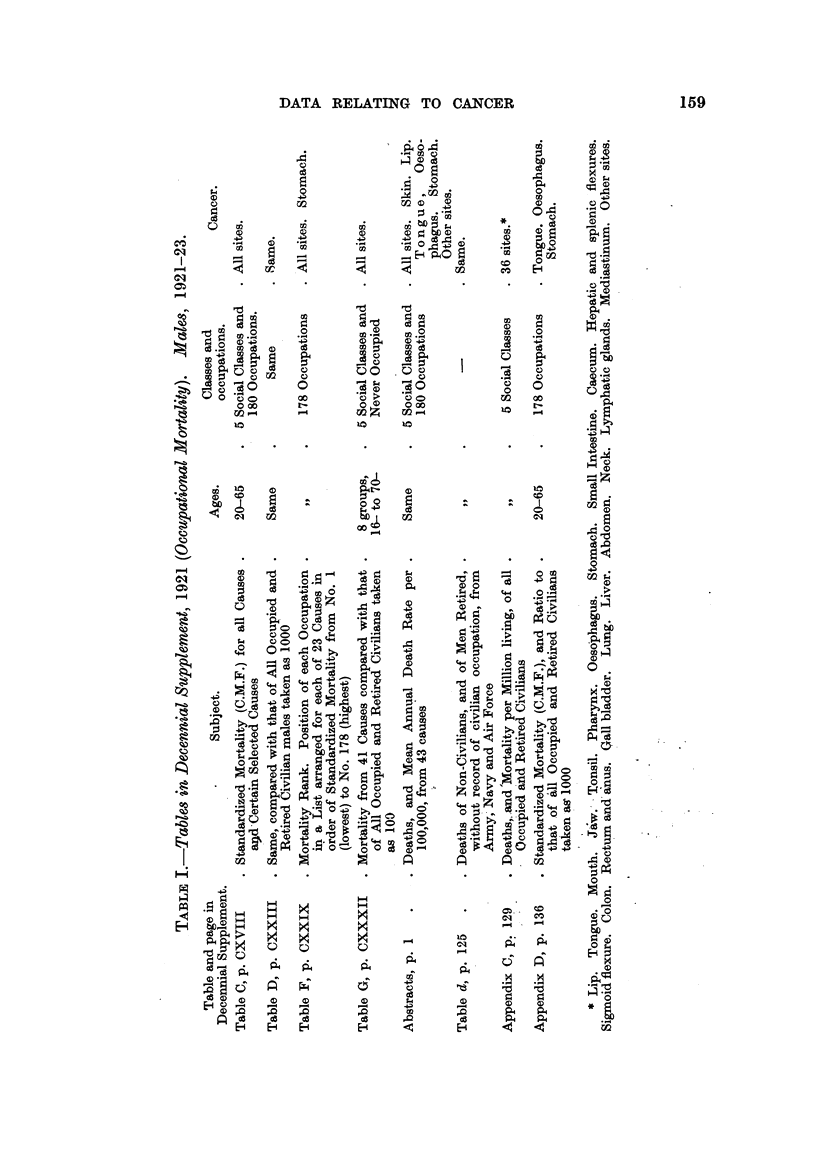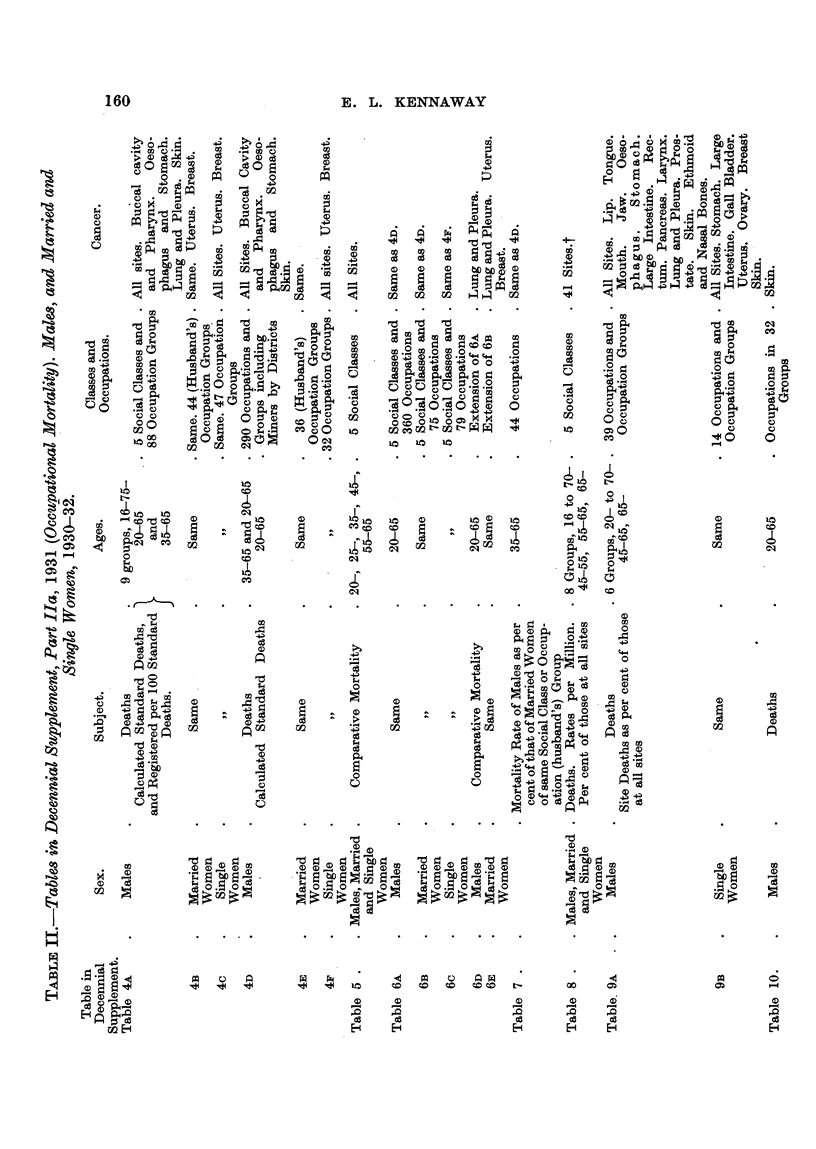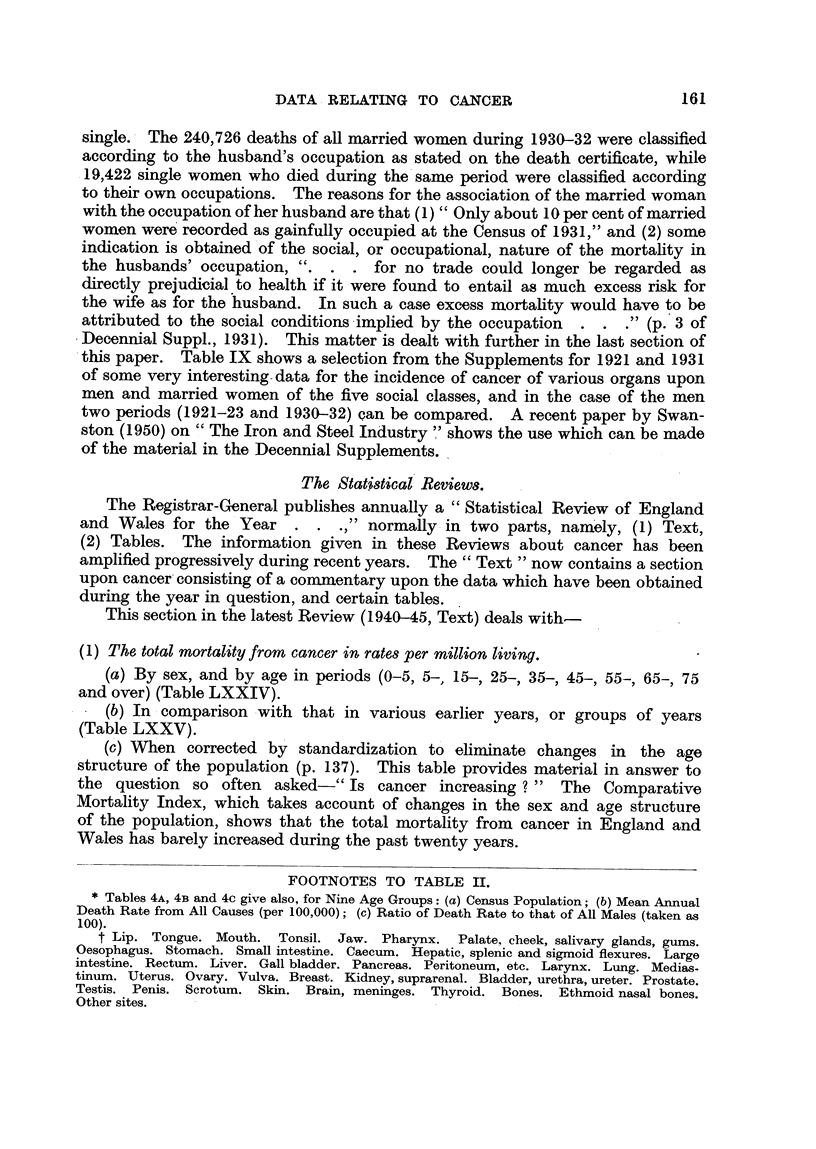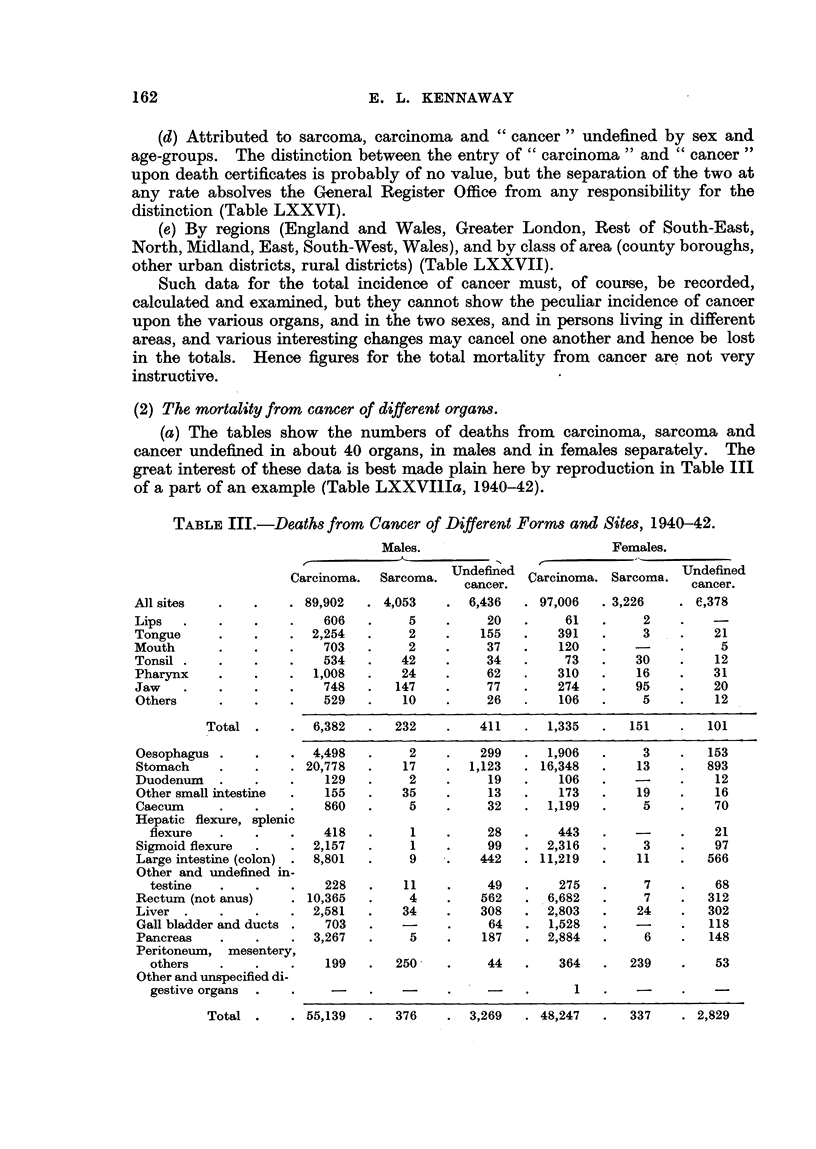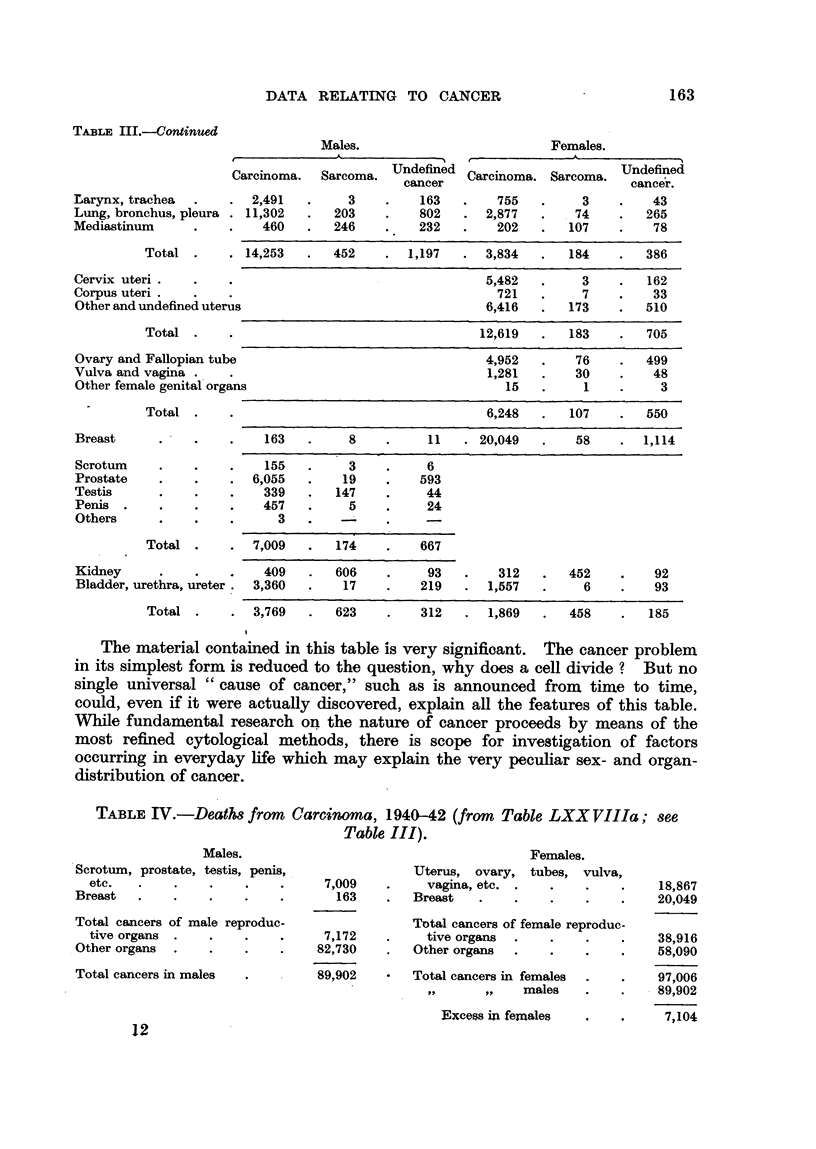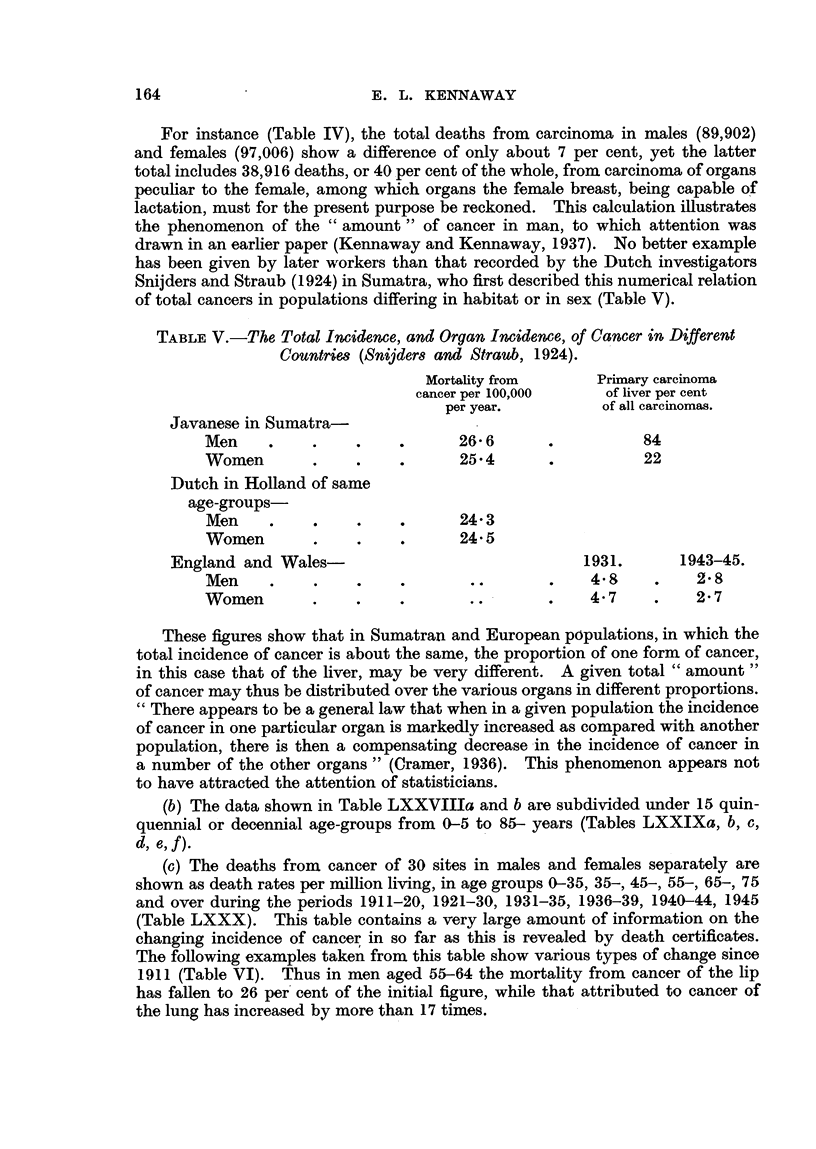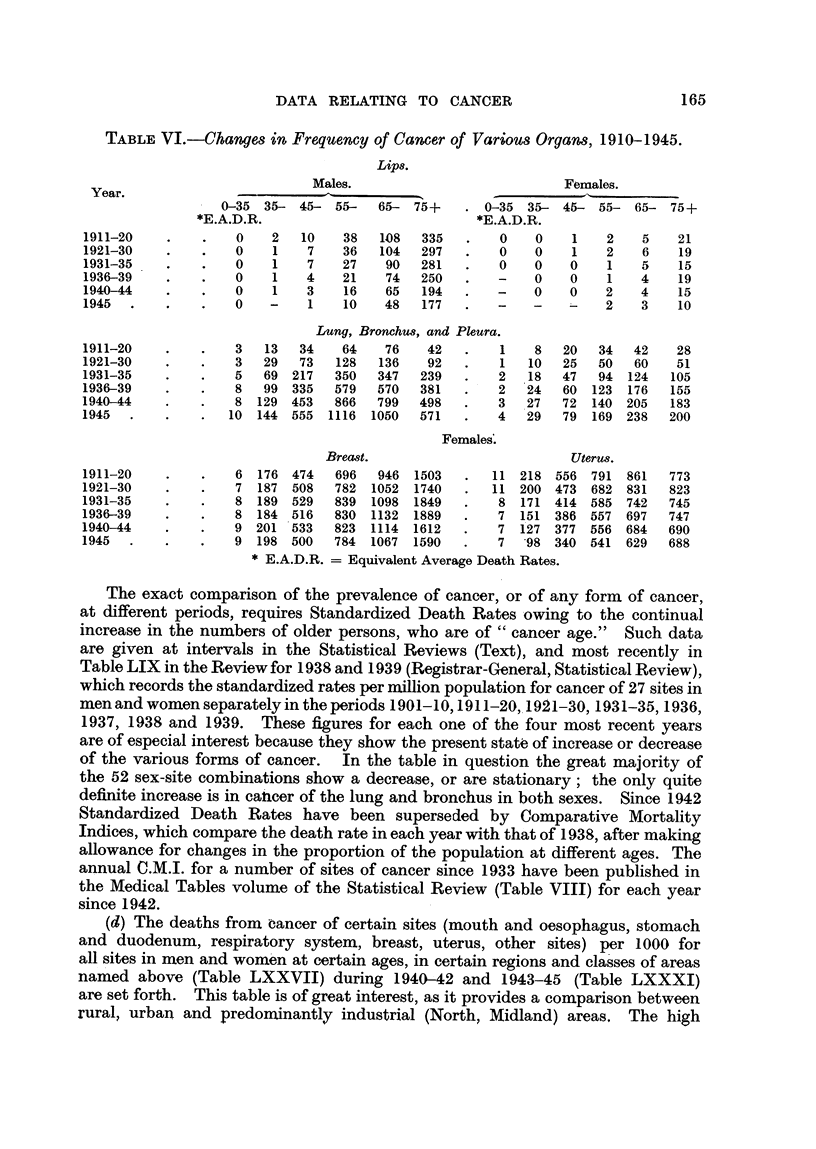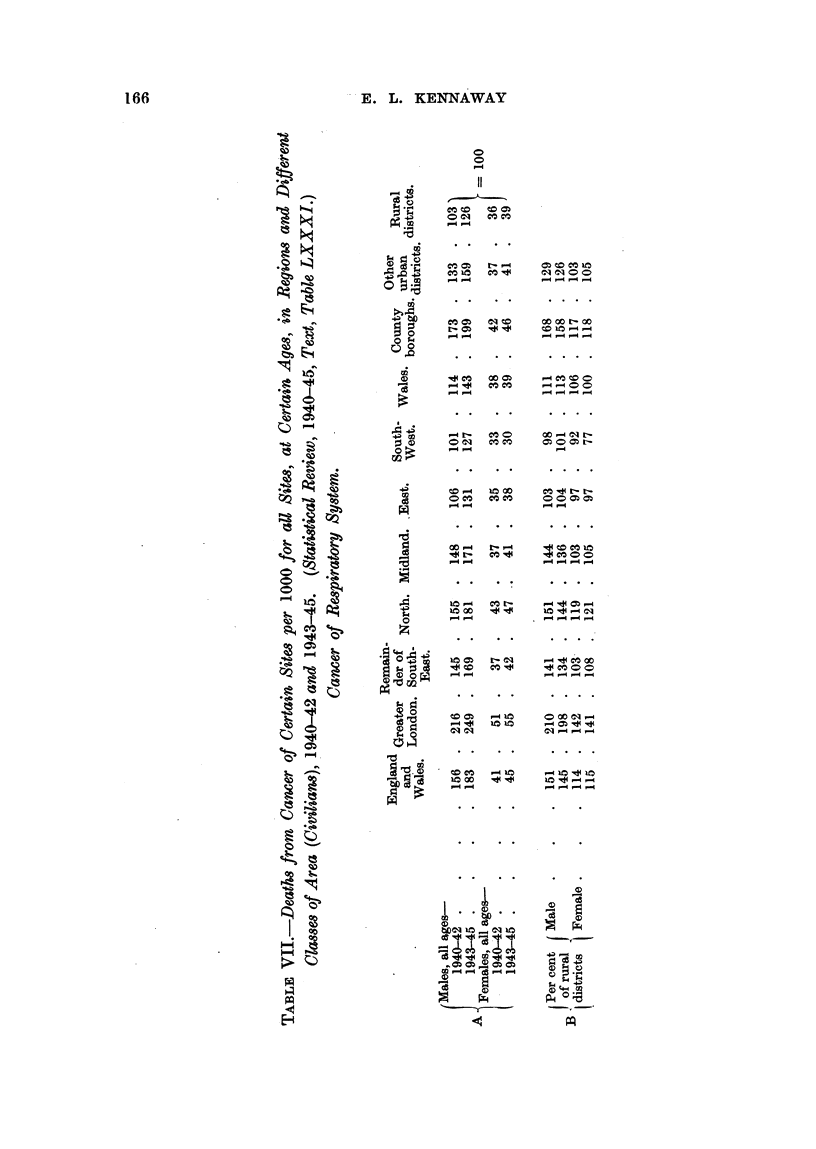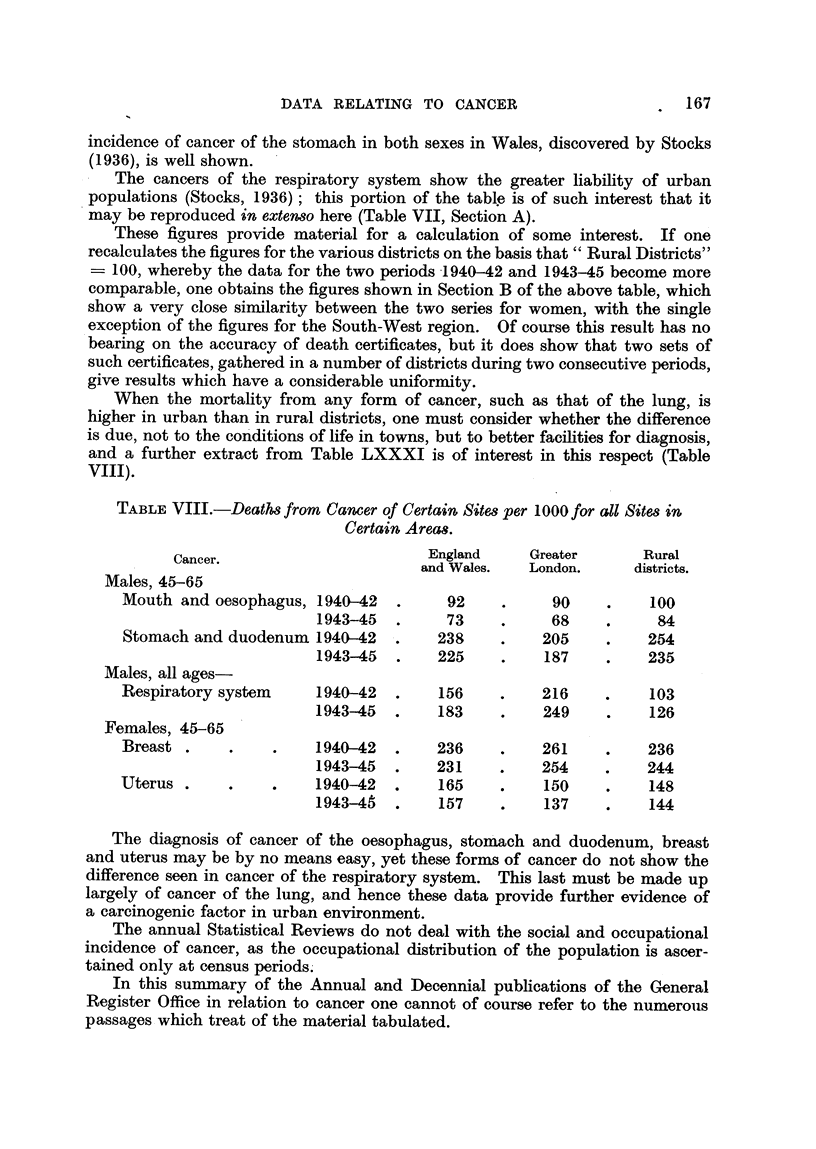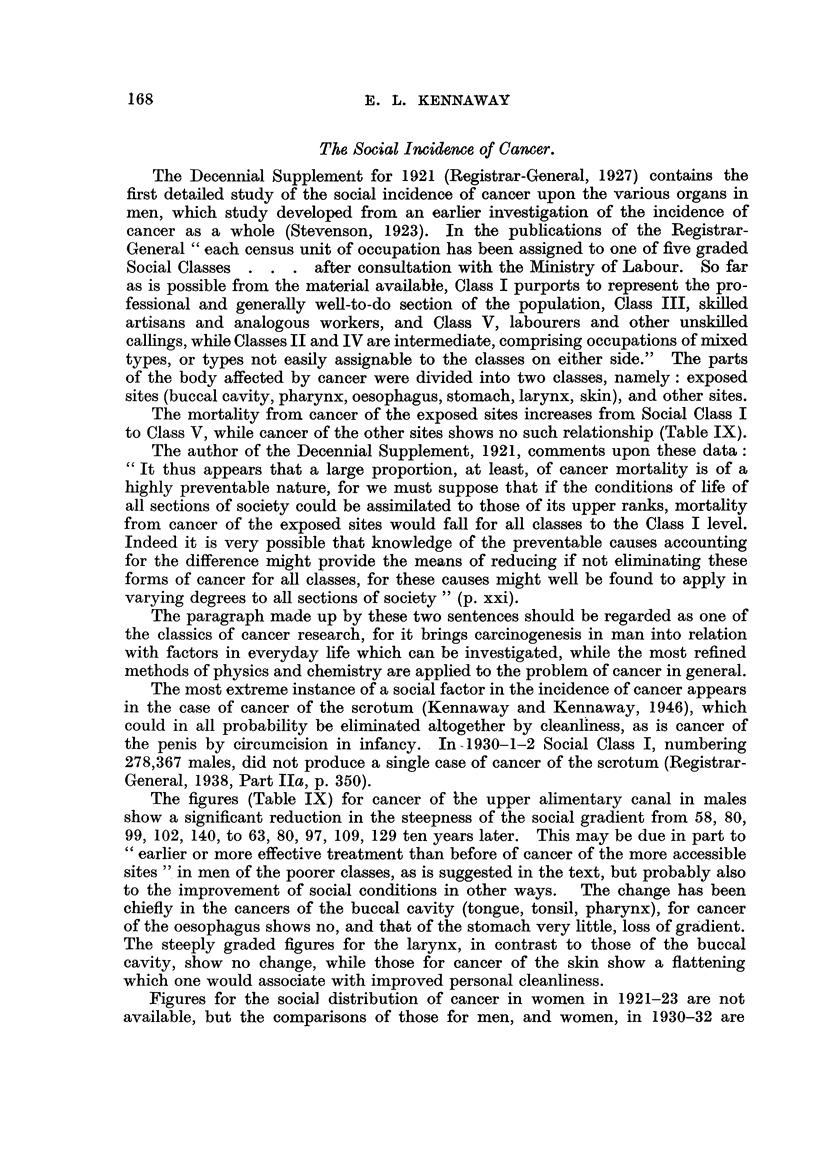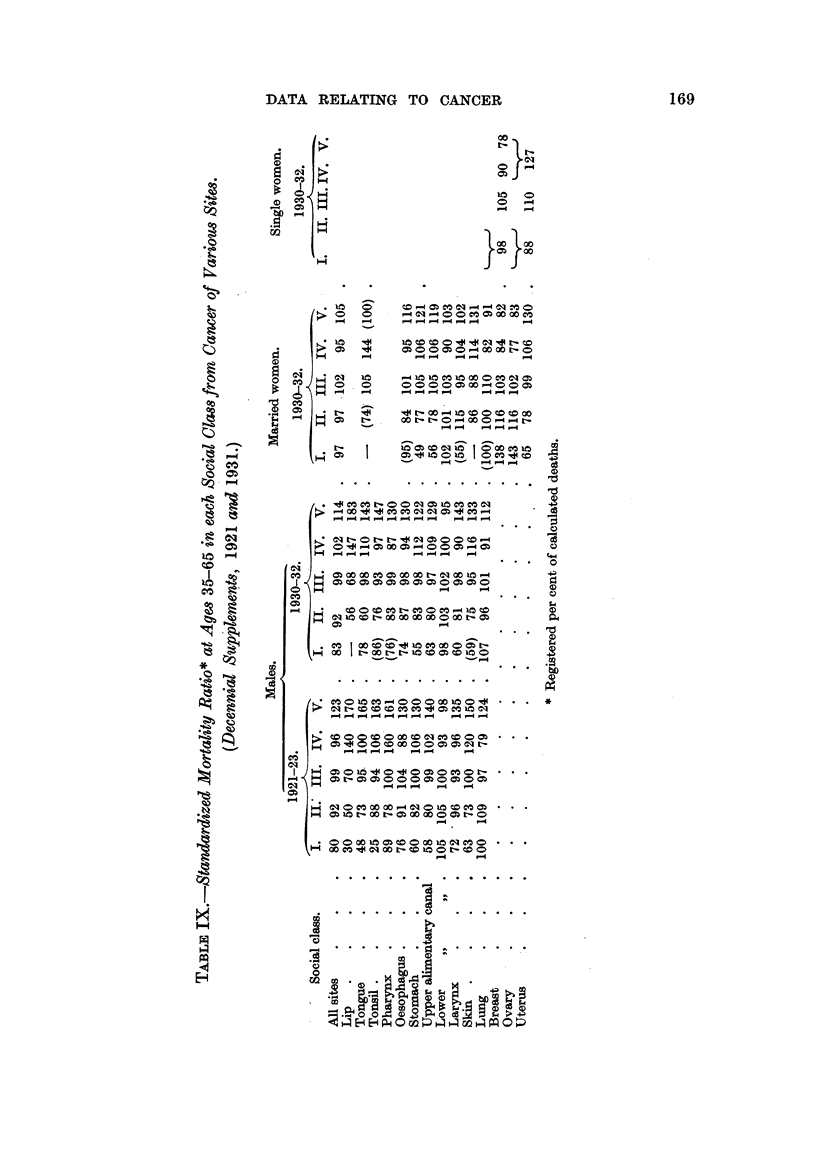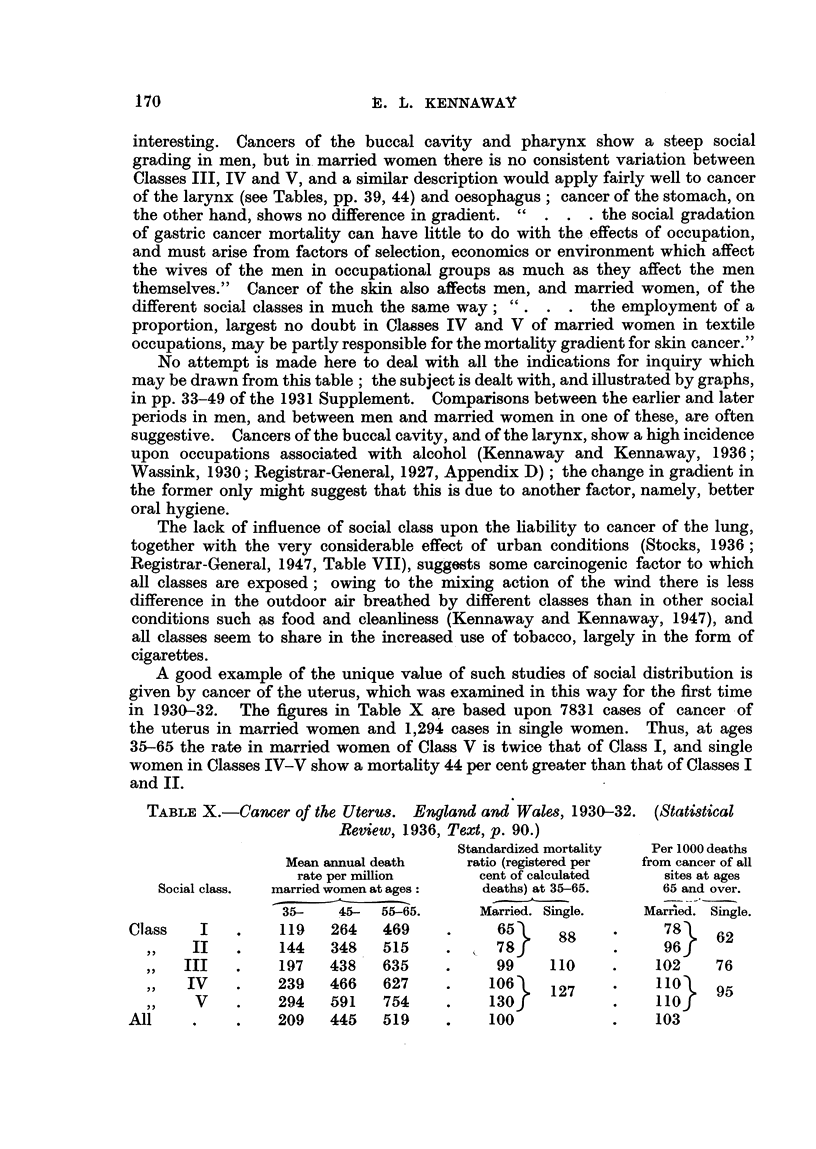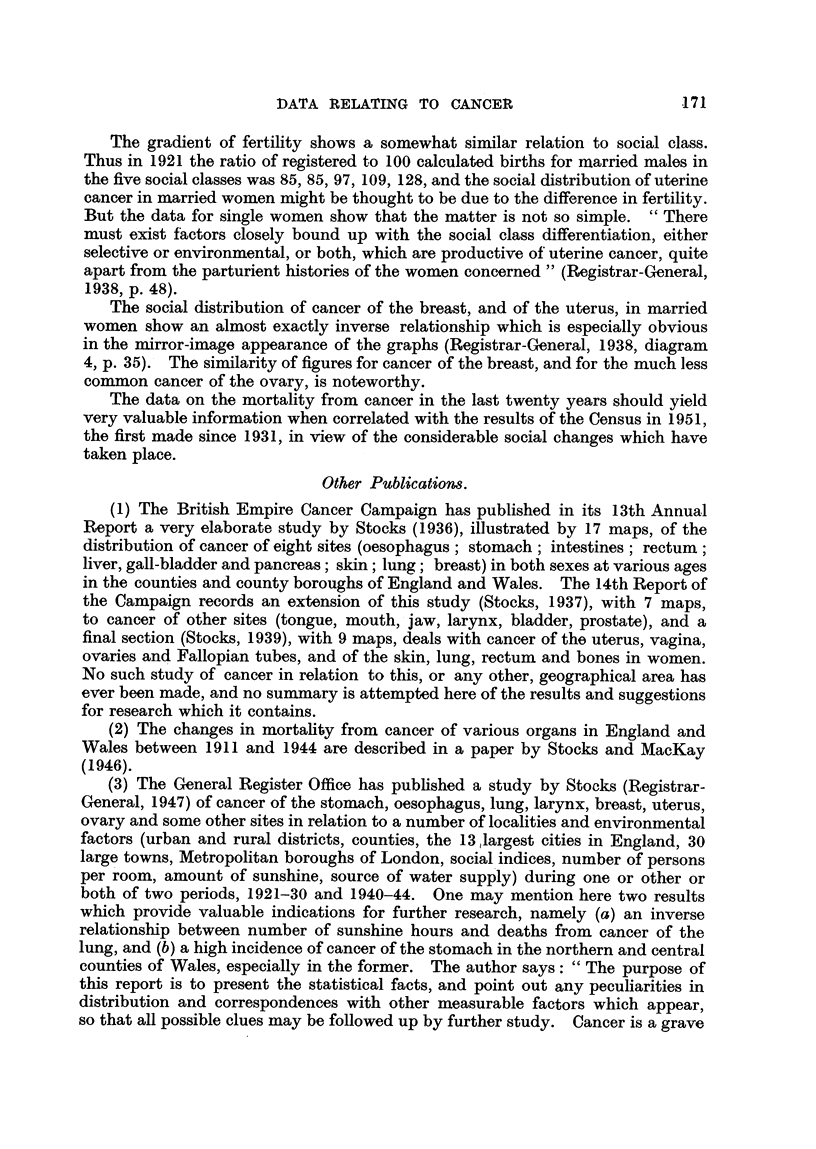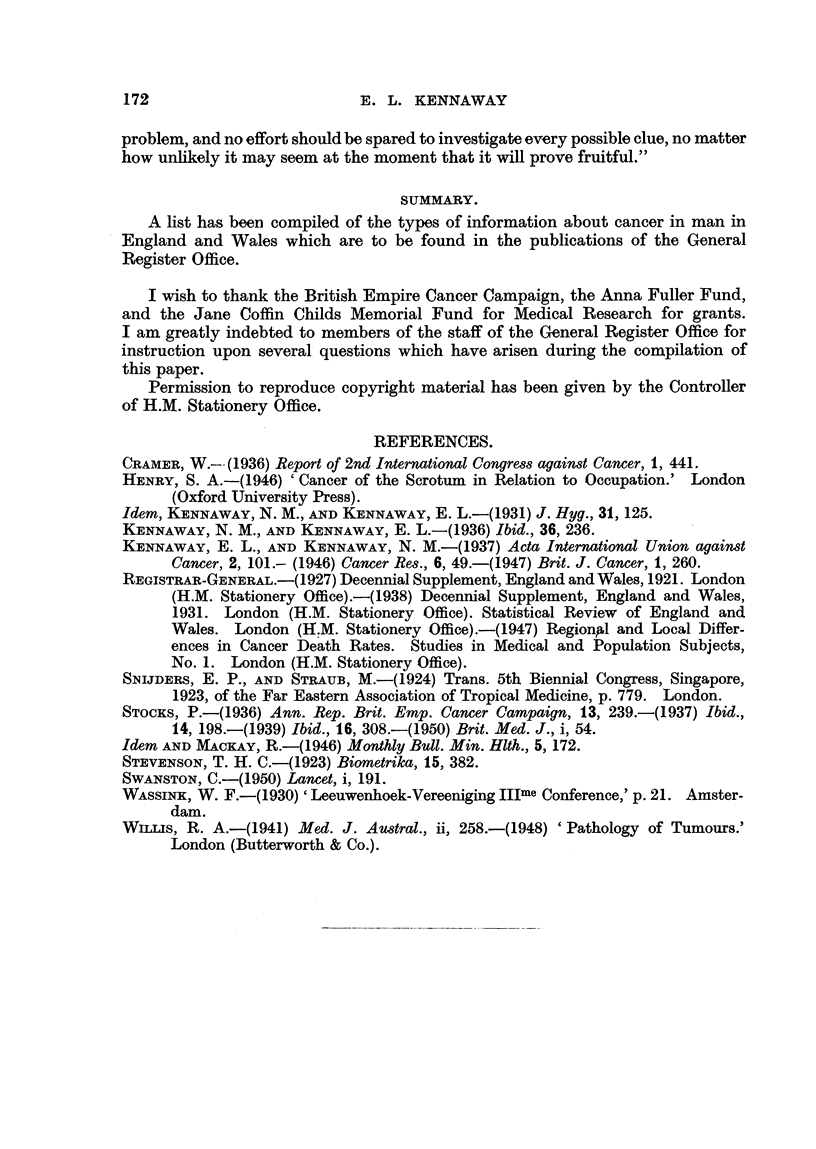# The Data Relating to Cancer in the Publications of the General Register Office

**DOI:** 10.1038/bjc.1950.16

**Published:** 1950-06

**Authors:** E. L. Kennaway


### THE DATA RELATING TO CANCER IN THE PUBLICATIONS OF THE GENERAL REGISTER OFFICE.

E. L. KENNAWAY.

*From the Pathological Department, St. Bartholomew's Hospital, London, E.C. 1.*

Received for publication March 10, 1950.

THE compilation of this paper was prompted by the idea that some investigators of cancer as it occurs in man do not know the valuable material which is contained in the publications of the General Register Office, while even those who are acquainted with this literature may find some use for a list of the collections of data about cancer which it contains. The paper is intended only to indicate the kind of information which is to be found ; no attempt is made to summarize the results.

The material dealt with in these publications consists of the death certificates from England and Wales, considered in association with the data on population, civil state, occupation and locality obtained at the Census of 1911, 1921 and 1931, together with population estimates for intervening and subsequent years. A death certificate gives the following particulars about the deceased person : name, sex, age, occupation, and date of death. Address at which death took place, and home address if different from this. District in which death was registered. Cause of death (primary and secondary). The certificate does not state, and is not intended to state, how long the deceased had followed the occupation named, and had lived at the home address given ; this information could be obtained only by inquiry into individual cases. The laborious nature of such inquiries, which are impracticable on any large scale, can be seen in the monograph by Henry (1946).

Many writers have emphasized the high proportion of errors in the assignment of the cause of death (Willis, 1941, 1948, pp. 66–78), and unpublished material by Willis quoted by Kennaway and Kennaway (1947). The sources of such errors have been discussed in two earlier papers (Henry, Kennaway and Kennaway, 1931 ; Kennaway and Kennaway, 1936). One must either recognize these errors and correct them in any way possible, or abandon any attempt to obtain information from these data. The General Register Office sends out every year ". . . some 14,000 inquiries for clarification or fuller information concerning causes " of death (Stocks, 1950). Some evidence on the uniformity of error in death certificates is given below.

*The Decennial Supplement (Occupational Mortality).*

The Decennial Supplements for 1921 and 1931 contain data collected at the Census of those years, and were published in 1927 and 1938 respectively (Registrar-General, 1927, 1938). They contain a large amount of tabulated material for periods adjacent to the Census, namely 1921–23 and 1930–32 ; the scope of those tables, in so far as they are concerned with cancer, is summarized in Tables I and II.

The Decennial Supplement for 1931 (Registrar-General, 1938) shows for the first time data for the social incidence of cancer upon women, both married and

TABLE I.—*Tables in Decennial Supplement*, 1921 (*Occupational Mortality*). *Males*, 1921–23.

| Table and page in Decennial Supplement. | Subject. | Ages. | Classes and occupations. | Cancer. |
|---|---|---|---|---|
| Table C, p. CXVIII | Standardized Mortality (C.M.F.) for all Causes and Certain Selected Causes | 20–65 | 5 Social Classes and 180 Occupations. | All sites. |
| Table D, p. CXXIII | Same, compared with that of All Occupied and Retired Civilian males taken as 1000 | Same | Same | Same. |
| Table F, p. CXXIX | Mortality Rank. Position of each Occupation in a List arranged for each of 23 Causes in order of Standardized Mortality from No. 1 (lowest) to No. 178 (highest) | ” | 178 Occupations | All sites. Stomach. |
| Table G, p. CXXXII | Mortality from 41 Causes compared with that of All Occupied and Retired Civilians taken as 100 | 8 groups, 16– to 70– | 5 Social Classes and Never Occupied | All sites. |
| Abstracts, p. 1 | Deaths, and Mean Annual Death Rate per 100,000, from 43 causes | Same | 5 Social Classes and 180 Occupations | All sites. Skin. Lip. Tongue, Oesophagus. Stomach. Other sites. |
| Table *d*, p. 125 | Deaths of Non-Civilians, and of Men Retired, without record of civilian occupation, from Army, Navy and Air Force | ” | — | Same. |
| Appendix C, p. 129 | Deaths, and Mortality per Million living, of all Occupied and Retired Civilians | ” | 5 Social Classes | 36 sites.* |
| Appendix D, p. 136 | Standardized Mortality (C.M.F.), and Ratio to that of all Occupied and Retired Civilians taken as 1000 | 20–65 | 178 Occupations | Tongue. Oesophagus. Stomach. |

* Lip. Tongue. Mouth. Jaw. Tonsil. Pharynx. Oesophagus. Stomach. Small Intestine. Caecum. Hepatic and splenic flexures. Sigmoid flexure. Colon. Rectum and anus. Gall bladder. Lung. Liver. Abdomen. Neck. Lymphatic glands. Mediastinum. Other sites.

TABLE II.—*Tables in Decennial Supplement, Part IIa*, 1931 (*Occupational Mortality*). *Males, and Married and Single Women*, 1930–32.

| Table in Decennial Supplement. | Sex. | Subject. | Ages. | Classes and Occupations. | Cancer. |
|---|---|---|---|---|---|
| Table 4A | Males | Deaths<br>Calculated Standard Deaths, and Registered per 100 Standard Deaths. | 9 groups, 16–75–<br>20–65 and 35–65 | 5 Social Classes and 88 Occupation Groups | All sites. Buccal cavity and Pharynx. Oesophagus and Stomach. Lung and Pleura. Skin. |
| 4B | Married Women | Same | Same | Same. 44 (Husband's) Occupation Groups | Same. Uterus. Breast. |
| 4C | Single Women | ,, | ,, | Same. 47 Occupation Groups | All Sites. Uterus. Breast. |
| 4D | Males | Deaths<br>Calculated Standard Deaths | 35–65 and 20–65<br>20–65 | 290 Occupations and Groups including Miners by Districts | All Sites. Buccal Cavity and Pharynx. Oesophagus and Stomach. Skin. |
| 4E | Married Women | Same | Same | 36 (Husband's) Occupation Groups | Same. |
| 4F | Single Women | ,, | ,, | 32 Occupation Groups | All sites. Uterus. Breast. |
| Table 5 | Males, Married and Single Women | Comparative Mortality | 20–, 25–, 35–, 45–, 55–65 | 5 Social Classes | All Sites. |
| Table 6A | Males | Same | 20–65 | 5 Social Classes and 360 Occupations | Same as 4D. |
| 6B | Married Women | ,, | Same | 5 Social Classes and 75 Occupations | Same as 4D. |
| 6C | Single Women | ,, | ,, | 5 Social Classes and 79 Occupations | Same as 4F. |
| 6D | Males | Comparative Mortality | 20–65 | Extension of 6A | Lung and Pleura. |
| 6E | Married Women | Same | Same | Extension of 6B | Lung and Pleura. Uterus. Breast. |
| Table 7 | | Mortality Rate of Males as per cent of that of Married Women of same Social Class or Occupation (husband's) Group | 35–65 | 44 Occupations | Same as 4D. |
| Table 8 | Males, Married and Single Women | Deaths. Rates per Million. Per cent of those at all sites | 8 Groups, 16 to 70–<br>45–55, 55–65, 65– | 5 Social Classes | 41 Sites.† |
| Table 9A | Males | Deaths<br>Site Deaths as per cent of those at all sites | 6 Groups, 20– to 70–<br>45–65, 65– | 39 Occupations and Occupation Groups | All Sites. Lip. Tongue. Mouth. Jaw. Oesophagus. Stomach. Large Intestine. Rectum. Pancreas. Larynx. Lung and Pleura. Prostate. Skin. Ethmoid and Nasal Bones. |
| 9B | Single Women | Same | Same | 14 Occupations and Occupation Groups | All Sites. Stomach. Large Intestine. Gall Bladder. Uterus. Ovary. Breast Skin. |
| Table 10 | Males | Deaths | 20–65 | Occupations in 32 Groups | Skin. |

single. The 240,726 deaths of all married women during 1930–32 were classified according to the husband's occupation as stated on the death certificate, while 19,422 single women who died during the same period were classified according to their own occupations. The reasons for the association of the married woman with the occupation of her husband are that (1) " Only about 10 per cent of married women were recorded as gainfully occupied at the Census of 1931," and (2) some indication is obtained of the social, or occupational, nature of the mortality in the husbands' occupation, ". . . for no trade could longer be regarded as directly prejudicial to health if it were found to entail as much excess risk for the wife as for the husband. In such a case excess mortality would have to be attributed to the social conditions implied by the occupation . . ." (p. 3 of Decennial Suppl., 1931). This matter is dealt with further in the last section of this paper. Table IX shows a selection from the Supplements for 1921 and 1931 of some very interesting data for the incidence of cancer of various organs upon men and married women of the five social classes, and in the case of the men two periods (1921–23 and 1930–32) can be compared. A recent paper by Swanston (1950) on " The Iron and Steel Industry " shows the use which can be made of the material in the Decennial Supplements.

*The Statistical Reviews.*

The Registrar-General publishes annually a " Statistical Review of England and Wales for the Year . . .," normally in two parts, namely, (1) Text, (2) Tables. The information given in these Reviews about cancer has been amplified progressively during recent years. The " Text " now contains a section upon cancer consisting of a commentary upon the data which have been obtained during the year in question, and certain tables.

This section in the latest Review (1940–45, Text) deals with—

(1) *The total mortality from cancer in rates per million living.*

(*a*) By sex, and by age in periods (0–5, 5–, 15–, 25–, 35–, 45–, 55–, 65–, 75 and over) (Table LXXIV).

(*b*) In comparison with that in various earlier years, or groups of years (Table LXXV).

(*c*) When corrected by standardization to eliminate changes in the age structure of the population (p. 137). This table provides material in answer to the question so often asked—" Is cancer increasing ? " The Comparative Mortality Index, which takes account of changes in the sex and age structure of the population, shows that the total mortality from cancer in England and Wales has barely increased during the past twenty years.

FOOTNOTES TO TABLE II.

* Tables 4A, 4B and 4C give also, for Nine Age Groups: (*a*) Census Population; (*b*) Mean Annual Death Rate from All Causes (per 100,000); (*c*) Ratio of Death Rate to that of All Males (taken as 100).

† Lip. Tongue. Mouth. Tonsil. Jaw. Pharynx. Palate, cheek, salivary glands, gums. Oesophagus. Stomach. Small intestine. Caecum. Hepatic, splenic and sigmoid flexures. Large intestine. Rectum. Liver. Gall bladder. Pancreas. Peritoneum, etc. Larynx. Lung. Mediastinum. Uterus. Ovary. Vulva. Breast. Kidney, suprarenal. Bladder, urethra, ureter. Prostate. Testis. Penis. Scrotum. Skin. Brain, meninges. Thyroid. Bones. Ethmoid nasal bones. Other sites.

(*d*) Attributed to sarcoma, carcinoma and " cancer " undefined by sex and age-groups. The distinction between the entry of " carcinoma " and " cancer " upon death certificates is probably of no value, but the separation of the two at any rate absolves the General Register Office from any responsibility for the distinction (Table LXXVI).

(*e*) By regions (England and Wales, Greater London, Rest of South-East, North, Midland, East, South-West, Wales), and by class of area (county boroughs, other urban districts, rural districts) (Table LXXVII).

Such data for the total incidence of cancer must, of course, be recorded, calculated and examined, but they cannot show the peculiar incidence of cancer upon the various organs, and in the two sexes, and in persons living in different areas, and various interesting changes may cancel one another and hence be lost in the totals. Hence figures for the total mortality from cancer are not very instructive.

(2) *The mortality from cancer of different organs.*

(*a*) The tables show the numbers of deaths from carcinoma, sarcoma and cancer undefined in about 40 organs, in males and in females separately. The great interest of these data is best made plain here by reproduction in Table III of a part of an example (Table LXXVIIIa, 1940–42).

TABLE III.—*Deaths from Cancer of Different Forms and Sites,* 1940–42.

| | Males. | | | Females. | | |
|---|---|---|---|---|---|---|
| | Carcinoma. | Sarcoma. | Undefined cancer. | Carcinoma. | Sarcoma. | Undefined cancer. |
| All sites | 89,902 | 4,053 | 6,436 | 97,006 | 3,226 | 6,378 |
| Lips | 606 | 5 | 20 | 61 | 2 | — |
| Tongue | 2,254 | 2 | 155 | 391 | 3 | 21 |
| Mouth | 703 | 2 | 37 | 120 | — | 5 |
| Tonsil | 534 | 42 | 34 | 73 | 30 | 12 |
| Pharynx | 1,008 | 24 | 62 | 310 | 16 | 31 |
| Jaw | 748 | 147 | 77 | 274 | 95 | 20 |
| Others | 529 | 10 | 26 | 106 | 5 | 12 |
| Total | 6,382 | 232 | 411 | 1,335 | 151 | 101 |
| Oesophagus | 4,498 | 2 | 299 | 1,906 | 3 | 153 |
| Stomach | 20,778 | 17 | 1,123 | 16,348 | 13 | 893 |
| Duodenum | 129 | 2 | 19 | 106 | — | 12 |
| Other small intestine | 155 | 35 | 13 | 173 | 19 | 16 |
| Caecum | 860 | 5 | 32 | 1,199 | 5 | 70 |
| Hepatic flexure, splenic flexure | 418 | 1 | 28 | 443 | — | 21 |
| Sigmoid flexure | 2,157 | 1 | 99 | 2,316 | 3 | 97 |
| Large intestine (colon) | 8,801 | 9 | 442 | 11,219 | 11 | 566 |
| Other and undefined intestine | 228 | 11 | 49 | 275 | 7 | 68 |
| Rectum (not anus) | 10,365 | 4 | 562 | 6,682 | 7 | 312 |
| Liver | 2,581 | 34 | 308 | 2,803 | 24 | 302 |
| Gall bladder and ducts | 703 | — | 64 | 1,528 | — | 118 |
| Pancreas | 3,267 | 5 | 187 | 2,884 | 6 | 148 |
| Peritoneum, mesentery, others | 199 | 250 | 44 | 364 | 239 | 53 |
| Other and unspecified digestive organs | — | — | — | 1 | — | — |
| Total | 55,139 | 376 | 3,269 | 48,247 | 337 | 2,829 |

TABLE III.—*Continued*

| | Males. | | | Females. | | |
|---|---|---|---|---|---|---|
| | Carcinoma. | Sarcoma. | Undefined cancer | Carcinoma. | Sarcoma. | Undefined cancer. |
| Larynx, trachea | 2,491 | 3 | 163 | 755 | 3 | 43 |
| Lung, bronchus, pleura | 11,302 | 203 | 802 | 2,877 | 74 | 265 |
| Mediastinum | 460 | 246 | 232 | 202 | 107 | 78 |
| Total | 14,253 | 452 | 1,197 | 3,834 | 184 | 386 |
| Cervix uteri | | | | 5,482 | 3 | 162 |
| Corpus uteri | | | | 721 | 7 | 33 |
| Other and undefined uterus | | | | 6,416 | 173 | 510 |
| Total | | | | 12,619 | 183 | 705 |
| Ovary and Fallopian tube | | | | 4,952 | 76 | 499 |
| Vulva and vagina | | | | 1,281 | 30 | 48 |
| Other female genital organs | | | | 15 | 1 | 3 |
| Total | | | | 6,248 | 107 | 550 |
| Breast | 163 | 8 | 11 | 20,049 | 58 | 1,114 |
| Scrotum | 155 | 3 | 6 | | | |
| Prostate | 6,055 | 19 | 593 | | | |
| Testis | 339 | 147 | 44 | | | |
| Penis | 457 | 5 | 24 | | | |
| Others | 3 | — | — | | | |
| Total | 7,009 | 174 | 667 | | | |
| Kidney | 409 | 606 | 93 | 312 | 452 | 92 |
| Bladder, urethra, ureter | 3,360 | 17 | 219 | 1,557 | 6 | 93 |
| Total | 3,769 | 623 | 312 | 1,869 | 458 | 185 |

The material contained in this table is very significant. The cancer problem in its simplest form is reduced to the question, why does a cell divide? But no single universal "cause of cancer," such as is announced from time to time, could, even if it were actually discovered, explain all the features of this table. While fundamental research on the nature of cancer proceeds by means of the most refined cytological methods, there is scope for investigation of factors occurring in everyday life which may explain the very peculiar sex- and organ-distribution of cancer.

TABLE IV.—*Deaths from Carcinoma,* 1940–42 (*from Table LXXVIIIa; see Table III*).

| Males. | | Females. | |
|---|---|---|---|
| Scrotum, prostate, testis, penis, etc. | 7,009 | Uterus, ovary, tubes, vulva, vagina, etc. | 18,867 |
| Breast | 163 | Breast | 20,049 |
| Total cancers of male reproductive organs | 7,172 | Total cancers of female reproductive organs | 38,916 |
| Other organs | 82,730 | Other organs | 58,090 |
| Total cancers in males | 89,902 | Total cancers in females | 97,006 |
| | | ,, ,, males | 89,902 |
| | | Excess in females | 7,104 |

For instance (Table IV), the total deaths from carcinoma in males (89,902) and females (97,006) show a difference of only about 7 per cent, yet the latter total includes 38,916 deaths, or 40 per cent of the whole, from carcinoma of organs peculiar to the female, among which organs the female breast, being capable of lactation, must for the present purpose be reckoned. This calculation illustrates the phenomenon of the " amount " of cancer in man, to which attention was drawn in an earlier paper (Kennaway and Kennaway, 1937). No better example has been given by later workers than that recorded by the Dutch investigators Snijders and Straub (1924) in Sumatra, who first described this numerical relation of total cancers in populations differing in habitat or in sex (Table V).

TABLE V.—*The Total Incidence, and Organ Incidence, of Cancer in Different Countries* (*Snijders and Straub*, 1924).

| | Mortality from cancer per 100,000 per year. | Primary carcinoma of liver per cent of all carcinomas. | |
|---|---|---|---|
| Javanese in Sumatra— | | | |
| Men | 26·6 | 84 | |
| Women | 25·4 | 22 | |
| Dutch in Holland of same age-groups— | | | |
| Men | 24·3 | | |
| Women | 24·5 | | |
| England and Wales— | | 1931. | 1943–45. |
| Men | .. | 4·8 | 2·8 |
| Women | .. | 4·7 | 2·7 |

These figures show that in Sumatran and European populations, in which the total incidence of cancer is about the same, the proportion of one form of cancer, in this case that of the liver, may be very different. A given total " amount " of cancer may thus be distributed over the various organs in different proportions. " There appears to be a general law that when in a given population the incidence of cancer in one particular organ is markedly increased as compared with another population, there is then a compensating decrease in the incidence of cancer in a number of the other organs " (Cramer, 1936). This phenomenon appears not to have attracted the attention of statisticians.

(*b*) The data shown in Table LXXVIII*a* and *b* are subdivided under 15 quinquennial or decennial age-groups from 0–5 to 85– years (Tables LXXIX*a*, *b*, *c*, *d*, *e*, *f*).

(*c*) The deaths from cancer of 30 sites in males and females separately are shown as death rates per million living, in age groups 0–35, 35–, 45–, 55–, 65–, 75 and over during the periods 1911–20, 1921–30, 1931–35, 1936–39, 1940–44, 1945 (Table LXXX). This table contains a very large amount of information on the changing incidence of cancer in so far as this is revealed by death certificates. The following examples taken from this table show various types of change since 1911 (Table VI). Thus in men aged 55–64 the mortality from cancer of the lip has fallen to 26 per cent of the initial figure, while that attributed to cancer of the lung has increased by more than 17 times.

TABLE VI.—*Changes in Frequency of Cancer of Various Organs*, 1910–1945.

| Year. | Males. | | | | | | Females. | | | | | |
|---|---|---|---|---|---|---|---|---|---|---|---|---|
| | 0–35 *E.A.D.R. | 35– | 45– | 55– | 65– | 75+ | 0–35 *E.A.D.R. | 35– | 45– | 55– | 65– | 75+ |
| | | | | | *Lips.* | | | | | | | |
| 1911–20 | 0 | 2 | 10 | 38 | 108 | 335 | 0 | 0 | 1 | 2 | 5 | 21 |
| 1921–30 | 0 | 1 | 7 | 36 | 104 | 297 | 0 | 0 | 1 | 2 | 6 | 19 |
| 1931–35 | 0 | 1 | 7 | 27 | 90 | 281 | 0 | 0 | 0 | 1 | 5 | 15 |
| 1936–39 | 0 | 1 | 4 | 21 | 74 | 250 | – | 0 | 0 | 1 | 4 | 19 |
| 1940–44 | 0 | 1 | 3 | 16 | 65 | 194 | – | 0 | 0 | 2 | 4 | 15 |
| 1945 | 0 | – | 1 | 10 | 48 | 177 | – | – | – | 2 | 3 | 10 |
| | | | | | *Lung, Bronchus, and Pleura.* | | | | | | | |
| 1911–20 | 3 | 13 | 34 | 64 | 76 | 42 | 1 | 8 | 20 | 34 | 42 | 28 |
| 1921–30 | 3 | 29 | 73 | 128 | 136 | 92 | 1 | 10 | 25 | 50 | 60 | 51 |
| 1931–35 | 5 | 69 | 217 | 350 | 347 | 239 | 2 | 18 | 47 | 94 | 124 | 105 |
| 1936–39 | 8 | 99 | 335 | 579 | 570 | 381 | 2 | 24 | 60 | 123 | 176 | 155 |
| 1940–44 | 8 | 129 | 453 | 866 | 799 | 498 | 3 | 27 | 72 | 140 | 205 | 183 |
| 1945 | 10 | 144 | 555 | 1116 | 1050 | 571 | 4 | 29 | 79 | 169 | 238 | 200 |
| | | | | | | Females. | | | | | | |
| | | | | *Breast.* | | | | | *Uterus.* | | | |
| 1911–20 | 6 | 176 | 474 | 696 | 946 | 1503 | 11 | 218 | 556 | 791 | 861 | 773 |
| 1921–30 | 7 | 187 | 508 | 782 | 1052 | 1740 | 11 | 200 | 473 | 682 | 831 | 823 |
| 1931–35 | 8 | 189 | 529 | 839 | 1098 | 1849 | 8 | 171 | 414 | 585 | 742 | 745 |
| 1936–39 | 8 | 184 | 516 | 830 | 1132 | 1889 | 7 | 151 | 386 | 557 | 697 | 747 |
| 1940–44 | 9 | 201 | 533 | 823 | 1114 | 1612 | 7 | 127 | 377 | 556 | 684 | 690 |
| 1945 | 9 | 198 | 500 | 784 | 1067 | 1590 | 7 | 98 | 340 | 541 | 629 | 688 |

* E.A.D.R. = Equivalent Average Death Rates.

The exact comparison of the prevalence of cancer, or of any form of cancer, at different periods, requires Standardized Death Rates owing to the continual increase in the numbers of older persons, who are of " cancer age." Such data are given at intervals in the Statistical Reviews (Text), and most recently in Table LIX in the Review for 1938 and 1939 (Registrar-General, Statistical Review), which records the standardized rates per million population for cancer of 27 sites in men and women separately in the periods 1901–10, 1911–20, 1921–30, 1931–35, 1936, 1937, 1938 and 1939. These figures for each one of the four most recent years are of especial interest because they show the present state of increase or decrease of the various forms of cancer. In the table in question the great majority of the 52 sex-site combinations show a decrease, or are stationary ; the only quite definite increase is in cancer of the lung and bronchus in both sexes. Since 1942 Standardized Death Rates have been superseded by Comparative Mortality Indices, which compare the death rate in each year with that of 1938, after making allowance for changes in the proportion of the population at different ages. The annual C.M.I. for a number of sites of cancer since 1933 have been published in the Medical Tables volume of the Statistical Review (Table VIII) for each year since 1942.

(*d*) The deaths from cancer of certain sites (mouth and oesophagus, stomach and duodenum, respiratory system, breast, uterus, other sites) per 1000 for all sites in men and women at certain ages, in certain regions and classes of areas named above (Table LXXVII) during 1940–42 and 1943–45 (Table LXXXI) are set forth. This table is of great interest, as it provides a comparison between rural, urban and predominantly industrial (North, Midland) areas. The high

TABLE VII.—*Deaths from Cancer of Certain Sites per* 1000 *for all Sites, at Certain Ages, in Regions and Different Classes of Area (Civilians),* 1940–42 *and* 1943–45. (*Statistical Review,* 1940–45, *Text, Table LXXXI.*)

*Cancer of Respiratory System.*

| | | England and Wales. | Greater London. | Remainder of South-East. | North. | Midland. | East. | South-West. | Wales. | County boroughs. | Other urban districts. | Rural districts. | |
|---|---|---|---|---|---|---|---|---|---|---|---|---|---|
| A | Males, all ages— | | | | | | | | | | | | |
| | 1940–42 | 156 | 216 | 145 | 155 | 148 | 106 | 101 | 114 | 173 | 133 | 103 | = 100 |
| | 1943–45 | 183 | 249 | 169 | 181 | 171 | 131 | 127 | 143 | 199 | 159 | 126 | = 100 |
| | Females, all ages— | | | | | | | | | | | | |
| | 1940–42 | 41 | 51 | 37 | 43 | 37 | 35 | 33 | 38 | 42 | 37 | 36 | = 100 |
| | 1943–45 | 45 | 55 | 42 | 47 | 41 | 38 | 30 | 39 | 46 | 41 | 39 | = 100 |
| B | Per cent of rural districts — Male | 151 | 210 | 141 | 151 | 144 | 103 | 98 | 111 | 168 | 129 | | |
| | | 145 | 198 | 134 | 144 | 136 | 104 | 101 | 113 | 158 | 126 | | |
| | Female | 114 | 142 | 103 | 119 | 103 | 97 | 92 | 106 | 117 | 103 | | |
| | | 115 | 141 | 108 | 121 | 105 | 97 | 77 | 100 | 118 | 105 | | |

incidence of cancer of the stomach in both sexes in Wales, discovered by Stocks (1936), is well shown.

The cancers of the respiratory system show the greater liability of urban populations (Stocks, 1936); this portion of the table is of such interest that it may be reproduced *in extenso* here (Table VII, Section A).

These figures provide material for a calculation of some interest. If one recalculates the figures for the various districts on the basis that "Rural Districts" = 100, whereby the data for the two periods 1940–42 and 1943–45 become more comparable, one obtains the figures shown in Section B of the above table, which show a very close similarity between the two series for women, with the single exception of the figures for the South-West region. Of course this result has no bearing on the accuracy of death certificates, but it does show that two sets of such certificates, gathered in a number of districts during two consecutive periods, give results which have a considerable uniformity.

When the mortality from any form of cancer, such as that of the lung, is higher in urban than in rural districts, one must consider whether the difference is due, not to the conditions of life in towns, but to better facilities for diagnosis, and a further extract from Table LXXXI is of interest in this respect (Table VIII).

TABLE VIII.—*Deaths from Cancer of Certain Sites per* 1000 *for all Sites in Certain Areas.*

| Cancer. | | England and Wales. | Greater London. | Rural districts. |
|---|---|---|---|---|
| Males, 45–65 | | | | |
| Mouth and oesophagus, | 1940–42 | 92 | 90 | 100 |
| | 1943–45 | 73 | 68 | 84 |
| Stomach and duodenum | 1940–42 | 238 | 205 | 254 |
| | 1943–45 | 225 | 187 | 235 |
| Males, all ages— | | | | |
| Respiratory system | 1940–42 | 156 | 216 | 103 |
| | 1943–45 | 183 | 249 | 126 |
| Females, 45–65 | | | | |
| Breast . . . | 1940–42 | 236 | 261 | 236 |
| | 1943–45 | 231 | 254 | 244 |
| Uterus . . . | 1940–42 | 165 | 150 | 148 |
| | 1943–45 | 157 | 137 | 144 |

The diagnosis of cancer of the oesophagus, stomach and duodenum, breast and uterus may be by no means easy, yet these forms of cancer do not show the difference seen in cancer of the respiratory system. This last must be made up largely of cancer of the lung, and hence these data provide further evidence of a carcinogenic factor in urban environment.

The annual Statistical Reviews do not deal with the social and occupational incidence of cancer, as the occupational distribution of the population is ascertained only at census periods.

In this summary of the Annual and Decennial publications of the General Register Office in relation to cancer one cannot of course refer to the numerous passages which treat of the material tabulated.

*The Social Incidence of Cancer.*

The Decennial Supplement for 1921 (Registrar-General, 1927) contains the first detailed study of the social incidence of cancer upon the various organs in men, which study developed from an earlier investigation of the incidence of cancer as a whole (Stevenson, 1923). In the publications of the Registrar-General " each census unit of occupation has been assigned to one of five graded Social Classes . . . after consultation with the Ministry of Labour. So far as is possible from the material available, Class I purports to represent the professional and generally well-to-do section of the population, Class III, skilled artisans and analogous workers, and Class V, labourers and other unskilled callings, while Classes II and IV are intermediate, comprising occupations of mixed types, or types not easily assignable to the classes on either side." The parts of the body affected by cancer were divided into two classes, namely : exposed sites (buccal cavity, pharynx, oesophagus, stomach, larynx, skin), and other sites.

The mortality from cancer of the exposed sites increases from Social Class I to Class V, while cancer of the other sites shows no such relationship (Table IX).

The author of the Decennial Supplement, 1921, comments upon these data : " It thus appears that a large proportion, at least, of cancer mortality is of a highly preventable nature, for we must suppose that if the conditions of life of all sections of society could be assimilated to those of its upper ranks, mortality from cancer of the exposed sites would fall for all classes to the Class I level. Indeed it is very possible that knowledge of the preventable causes accounting for the difference might provide the means of reducing if not eliminating these forms of cancer for all classes, for these causes might well be found to apply in varying degrees to all sections of society " (p. xxi).

The paragraph made up by these two sentences should be regarded as one of the classics of cancer research, for it brings carcinogenesis in man into relation with factors in everyday life which can be investigated, while the most refined methods of physics and chemistry are applied to the problem of cancer in general.

The most extreme instance of a social factor in the incidence of cancer appears in the case of cancer of the scrotum (Kennaway and Kennaway, 1946), which could in all probability be eliminated altogether by cleanliness, as is cancer of the penis by circumcision in infancy. In 1930–1–2 Social Class I, numbering 278,367 males, did not produce a single case of cancer of the scrotum (Registrar-General, 1938, Part II*a*, p. 350).

The figures (Table IX) for cancer of the upper alimentary canal in males show a significant reduction in the steepness of the social gradient from 58, 80, 99, 102, 140, to 63, 80, 97, 109, 129 ten years later. This may be due in part to " earlier or more effective treatment than before of cancer of the more accessible sites " in men of the poorer classes, as is suggested in the text, but probably also to the improvement of social conditions in other ways. The change has been chiefly in the cancers of the buccal cavity (tongue, tonsil, pharynx), for cancer of the oesophagus shows no, and that of the stomach very little, loss of gradient. The steeply graded figures for the larynx, in contrast to those of the buccal cavity, show no change, while those for cancer of the skin show a flattening which one would associate with improved personal cleanliness.

Figures for the social distribution of cancer in women in 1921–23 are not available, but the comparisons of those for men, and women, in 1930–32 are

TABLE IX.—*Standardized Mortality Ratio* at Ages 35–65 in each Social Class from Cancer of Various Sites.*

(*Decennial Supplements*, 1921 *and* 1931.)

| | Males. | | | | | | | | | | Married women. | | | | | Single women. | | | | |
|---|---|---|---|---|---|---|---|---|---|---|---|---|---|---|---|---|---|---|---|---|
| | 1921–23. | | | | | 1930–32. | | | | | 1930–32. | | | | | 1930–32. | | | | |
| Social class. | I. | II. | III. | IV. | V. | I. | II. | III. | IV. | V. | I. | II. | III. | IV. | V. | I. | II. | III. | IV. | V. |
| All sites | 80 | 92 | 99 | 96 | 123 | 83 | 92 | 99 | 102 | 114 | 97 | 97 | 102 | 95 | 105 | | | | | |
| Lip | 30 | 50 | 70 | 140 | 170 | — | 56 | 68 | 147 | 183 | | | | | | | | | | |
| Tongue | 48 | 73 | 95 | 100 | 165 | 78 | 60 | 98 | 110 | 143 | — | (74) | 105 | 144 | (100) | | | | | |
| Tonsil | 25 | 88 | 94 | 106 | 163 | (86) | 76 | 93 | 97 | 147 | | | | | | | | | | |
| Pharynx | 89 | 78 | 100 | 160 | 161 | (76) | 83 | 99 | 87 | 130 | | | | | | | | | | |
| Oesophagus | 76 | 91 | 104 | 88 | 130 | 74 | 87 | 98 | 94 | 130 | (95) | 84 | 101 | 95 | 116 | | | | | |
| Stomach | 60 | 82 | 100 | 106 | 130 | 55 | 83 | 98 | 112 | 122 | 49 | 77 | 105 | 106 | 121 | | | | | |
| Upper alimentary canal | 58 | 80 | 99 | 102 | 140 | 63 | 80 | 97 | 109 | 129 | 56 | 78 | 105 | 106 | 119 | | | | | |
| Lower „ „ | 105 | 105 | 100 | 93 | 98 | 98 | 103 | 102 | 100 | 95 | 102 | 101 | 103 | 90 | 103 | | | | | |
| Larynx | 72 | 96 | 93 | 96 | 135 | 60 | 81 | 98 | 90 | 143 | (55) | 115 | 95 | 104 | 102 | | | | | |
| Skin | 63 | 73 | 100 | 120 | 150 | (59) | 75 | 95 | 116 | 133 | — | 86 | 88 | 114 | 131 | | | | | |
| Lung | 100 | 109 | 97 | 79 | 124 | 107 | 96 | 101 | 91 | 112 | (100) | 100 | 110 | 82 | 91 | | | | | |
| Breast | . | . | . | . | . | . | . | . | . | . | 138 | 116 | 103 | 84 | 82 | 98 (I–II) | | 105 | 90 | 78 |
| Ovary | . | . | . | . | . | . | . | . | . | . | 143 | 116 | 102 | 77 | 83 | | | | | |
| Uterus | . | . | . | . | . | . | . | . | . | . | 65 | 78 | 99 | 106 | 130 | 88 (I–II) | | 110 | 127 (IV–V) | |

* Registered per cent of calculated deaths.

interesting. Cancers of the buccal cavity and pharynx show a steep social grading in men, but in married women there is no consistent variation between Classes III, IV and V, and a similar description would apply fairly well to cancer of the larynx (see Tables, pp. 39, 44) and oesophagus ; cancer of the stomach, on the other hand, shows no difference in gradient. " . . . the social gradation of gastric cancer mortality can have little to do with the effects of occupation, and must arise from factors of selection, economics or environment which affect the wives of the men in occupational groups as much as they affect the men themselves." Cancer of the skin also affects men, and married women, of the different social classes in much the same way ; " . . . the employment of a proportion, largest no doubt in Classes IV and V of married women in textile occupations, may be partly responsible for the mortality gradient for skin cancer."

No attempt is made here to deal with all the indications for inquiry which may be drawn from this table ; the subject is dealt with, and illustrated by graphs, in pp. 33–49 of the 1931 Supplement. Comparisons between the earlier and later periods in men, and between men and married women in one of these, are often suggestive. Cancers of the buccal cavity, and of the larynx, show a high incidence upon occupations associated with alcohol (Kennaway and Kennaway, 1936; Wassink, 1930; Registrar-General, 1927, Appendix D) ; the change in gradient in the former only might suggest that this is due to another factor, namely, better oral hygiene.

The lack of influence of social class upon the liability to cancer of the lung, together with the very considerable effect of urban conditions (Stocks, 1936 ; Registrar-General, 1947, Table VII), suggests some carcinogenic factor to which all classes are exposed ; owing to the mixing action of the wind there is less difference in the outdoor air breathed by different classes than in other social conditions such as food and cleanliness (Kennaway and Kennaway, 1947), and all classes seem to share in the increased use of tobacco, largely in the form of cigarettes.

A good example of the unique value of such studies of social distribution is given by cancer of the uterus, which was examined in this way for the first time in 1930–32. The figures in Table X are based upon 7831 cases of cancer of the uterus in married women and 1,294 cases in single women. Thus, at ages 35–65 the rate in married women of Class V is twice that of Class I, and single women in Classes IV–V show a mortality 44 per cent greater than that of Classes I and II.

TABLE X.—*Cancer of the Uterus. England and Wales*, 1930–32. (*Statistical Review*, 1936, *Text*, *p.* 90.)

| Social class. | Mean annual death rate per million married women at ages : 35– | 45– | 55–65. | Standardized mortality ratio (registered per cent of calculated deaths) at 35–65. Married. | Single. | Per 1000 deaths from cancer of all sites at ages 65 and over. Married. | Single. |
|---|---|---|---|---|---|---|---|
| Class I | 119 | 264 | 469 | 65 | 88 (I–II) | 78 | 62 (I–II) |
| ,, II | 144 | 348 | 515 | 78 | | 96 | |
| ,, III | 197 | 438 | 635 | 99 | 110 | 102 | 76 |
| ,, IV | 239 | 466 | 627 | 106 | 127 (IV–V) | 110 | 95 (IV–V) |
| ,, V | 294 | 591 | 754 | 130 | | 110 | |
| All | 209 | 445 | 519 | 100 | | 103 | |

The gradient of fertility shows a somewhat similar relation to social class. Thus in 1921 the ratio of registered to 100 calculated births for married males in the five social classes was 85, 85, 97, 109, 128, and the social distribution of uterine cancer in married women might be thought to be due to the difference in fertility. But the data for single women show that the matter is not so simple. "There must exist factors closely bound up with the social class differentiation, either selective or environmental, or both, which are productive of uterine cancer, quite apart from the parturient histories of the women concerned" (Registrar-General, 1938, p. 48).

The social distribution of cancer of the breast, and of the uterus, in married women show an almost exactly inverse relationship which is especially obvious in the mirror-image appearance of the graphs (Registrar-General, 1938, diagram 4, p. 35). The similarity of figures for cancer of the breast, and for the much less common cancer of the ovary, is noteworthy.

The data on the mortality from cancer in the last twenty years should yield very valuable information when correlated with the results of the Census in 1951, the first made since 1931, in view of the considerable social changes which have taken place.

*Other Publications.*

(1) The British Empire Cancer Campaign has published in its 13th Annual Report a very elaborate study by Stocks (1936), illustrated by 17 maps, of the distribution of cancer of eight sites (oesophagus ; stomach ; intestines ; rectum ; liver, gall-bladder and pancreas ; skin ; lung ; breast) in both sexes at various ages in the counties and county boroughs of England and Wales. The 14th Report of the Campaign records an extension of this study (Stocks, 1937), with 7 maps, to cancer of other sites (tongue, mouth, jaw, larynx, bladder, prostate), and a final section (Stocks, 1939), with 9 maps, deals with cancer of the uterus, vagina, ovaries and Fallopian tubes, and of the skin, lung, rectum and bones in women. No such study of cancer in relation to this, or any other, geographical area has ever been made, and no summary is attempted here of the results and suggestions for research which it contains.

(2) The changes in mortality from cancer of various organs in England and Wales between 1911 and 1944 are described in a paper by Stocks and MacKay (1946).

(3) The General Register Office has published a study by Stocks (Registrar-General, 1947) of cancer of the stomach, oesophagus, lung, larynx, breast, uterus, ovary and some other sites in relation to a number of localities and environmental factors (urban and rural districts, counties, the 13 largest cities in England, 30 large towns, Metropolitan boroughs of London, social indices, number of persons per room, amount of sunshine, source of water supply) during one or other or both of two periods, 1921–30 and 1940–44. One may mention here two results which provide valuable indications for further research, namely (*a*) an inverse relationship between number of sunshine hours and deaths from cancer of the lung, and (*b*) a high incidence of cancer of the stomach in the northern and central counties of Wales, especially in the former. The author says: "The purpose of this report is to present the statistical facts, and point out any peculiarities in distribution and correspondences with other measurable factors which appear, so that all possible clues may be followed up by further study. Cancer is a grave problem, and no effort should be spared to investigate every possible clue, no matter how unlikely it may seem at the moment that it will prove fruitful."

SUMMARY.

A list has been compiled of the types of information about cancer in man in England and Wales which are to be found in the publications of the General Register Office.

I wish to thank the British Empire Cancer Campaign, the Anna Fuller Fund, and the Jane Coffin Childs Memorial Fund for Medical Research for grants. I am greatly indebted to members of the staff of the General Register Office for instruction upon several questions which have arisen during the compilation of this paper.

Permission to reproduce copyright material has been given by the Controller of H.M. Stationery Office.

### REFERENCES.

CRAMER, W.—(1936) *Report of 2nd International Congress against Cancer*, **1**, 441.
HENRY, S. A.—(1946) 'Cancer of the Scrotum in Relation to Occupation.' London (Oxford University Press).
*Idem*, KENNAWAY, N. M., AND KENNAWAY, E. L.—(1931) *J. Hyg.*, **31**, 125.
KENNAWAY, N. M., AND KENNAWAY, E. L.—(1936) *Ibid.*, **36**, 236.
KENNAWAY, E. L., AND KENNAWAY, N. M.—(1937) *Acta International Union against Cancer*, **2**, 101.– (1946) *Cancer Res.*, **6**, 49.—(1947) *Brit. J. Cancer*, **1**, 260.
REGISTRAR-GENERAL.—(1927) Decennial Supplement, England and Wales, 1921. London (H.M. Stationery Office).—(1938) Decennial Supplement, England and Wales, 1931. London (H.M. Stationery Office). Statistical Review of England and Wales. London (H.M. Stationery Office).—(1947) Regional and Local Differences in Cancer Death Rates. Studies in Medical and Population Subjects, No. 1. London (H.M. Stationery Office).
SNIJDERS, E. P., AND STRAUB, M.—(1924) Trans. 5th Biennial Congress, Singapore, 1923, of the Far Eastern Association of Tropical Medicine, p. 779. London.
STOCKS, P.—(1936) *Ann. Rep. Brit. Emp. Cancer Campaign*, **13**, 239.—(1937) *Ibid.*, **14**, 198.—(1939) *Ibid.*, **16**, 308.—(1950) *Brit. Med. J.*, i, 54.
*Idem* AND MACKAY, R.—(1946) *Monthly Bull. Min. Hlth.*, **5**, 172.
STEVENSON, T. H. C.—(1923) *Biometrika*, **15**, 382.
SWANSTON, C.—(1950) *Lancet*, i, 191.
WASSINK, W. F.—(1930) 'Leeuwenhoek-Vereeniging III$^{me}$ Conference,' p. 21. Amsterdam.
WILLIS, R. A.—(1941) *Med. J. Austral.*, ii, 258.—(1948) 'Pathology of Tumours.' London (Butterworth & Co.).